# Appendicitis Complicating Pregnancy

**Published:** 1917-02

**Authors:** Aime’ Paul Heineck

**Affiliations:** Professor of Surgery, Chicago College of Medicine and Surgery Chicago


### Journal-Record of Medicine

Successor to Atlanta Medical and Surgical Journal, Established 1855
and Southern Medical Record, Established 1870

**OWNED BY THE ATLANTA MEDICAL JOURNAL COMPANY**

PUBLISHED MONTHLY

*Official Organ Fulton County Medical Society, State Examing Board, Atlanta, Birmingham and Atlantic Railroad Surgeon's Association, Chattahoochee Valley Medical and Surgical Association, Etc.*

A. W. STIRLING, M. D., C. M., D. P. H.; J. S. HURT, B. Ph., M.D.
GEO. M. NILES, M. D., W. J. LOVE, M. D., (Ala.); Associate Editors

E. W. ALLEN, Business Manager

**COLLABORATORS**

W. F. WESTMORELAND, M. D., General Surgery
F. W. McRAE, M. D., Abdominal Surgery
ALLEN H. BUNCE, Medical Societies
E. B. BLOCK, M. D., Diseases of the Nervous System
MICHAEL HOKE, M. D., Orthopedic Surgery
CYRUS W. STRICKLER, M. D., Legal Medicine and Medical Legislation
E. C. DAVIS, A. B., M. D., Obstetrics
E. G. JONES, A. B., M. D.,. Gynecology
ALLEN H. BUNCE, M. D., Pathology and Bacteriology
R. T. DORSEY, JR., B. S., M. D., Medicine
L. M. GAINES, A. B., M. D., Internal Medicine
GEO. C. MIZELL, M. D., Diseases of the Stomach and Intestines
L. B. CLARKE, M. D., Pediatrics
EDGAR PAULLIN, M. D., Opsonic Medicine
THEODORE TOEPEL, M. D., Mechano Therapy
E. G. BALLENGER, M. D., Diseases of the Genito-Urinary Organs

VOL. LXIII. ATLANTA, GA., FEBRUARY, 1917. NO. 11

### APPENDICITIS COMPLICATING PREGNANCY.

**By**

**Aime' Paul Heineck, M. D.**

**Professor of Surgery, Chicago College of Medicine and Surgery Chicago.**

Appendicitis attacks all ages and both sexes· Though distinctly a surgical disease, it is also of great practical interest to gynecologists, obstetricians and general practitioners.

The frequency of appendicitis in the female pregnant or non-pregnant is under-estimated and its significance not fully appreciated. It is often overlooked, misdiagnosed, and therefore improperly treated· The autopsy findings often bring the first intimation of the true cause of the clinical picture.

To serve our fellow practitioners, we collected, analyzed and studied the original reports of all the operated cases of appendicitis occurring during pregnancy, that are to be found in the French, English and German medical literature from 1900 to 1915, inclusive, and also some unpublished personal cases. Cases reported with insufficient data were not considered·

The subject will be discussed under the following subheads:

1. Incidence.
2. Etiology.
3. Combined appendicitis and extra-uterine Pregnancy.
4. Pathology.
5. Coexisting conditions.
   Influence of pregnancy upon appendicitis.
   Influence of appendicitis upon pregnancy.
6. Diagnosis
7. Differential diagnosis.
   (a) Maternal.
   (b) Fetal.
8· Prognosis.
9. Treatment·
   (a) Prophylaxis.
   (b) Indication for operation.
   (c) Operative.
10. Post operative sequelae.
11. Summary.

**Incidence.**

During the child-bearing age, woman is at no time exempt from attacks of appendicitis. In forty-six of our selected cases, the age is not stated. The remaining patients were at time of operation:

Under 18 years ____________________________3 cases.
18—20 years inclusive ____________________13 cases.
21—25 years inclusive ____________________33 cases.
26—30 years inclusive ____________________42 cases.
31—35 years inclusive ____________________23 cases.
36—40 years inclusive ____________________12 cases.
One patient forty-two years.

The condition occurs in primiparae and multiparae; in first early and late pregnancies; in single and twin pregnancies.

Appendicitis can coexist with other disease processes to which it may be primary, secondary or coincidental.

In the cases forming the basis of this article, there are noted thirty primiparae, twenty deutiparae, thirty-seven multiparae.

The number of previous pregnancies if there were any, is not stated in eighty-three cases. Appendicitis occurs at all periods of gestation. In some cases, the disease antedated pregnancy; some cases were operated early with reference to onset of symptoms; some late. It is recorded that operation was indicated and performed:

During the first three months of gestation__40 times.
From 4—6 months, inclusive______________60 times.
From 7—9 months, inclusive______________28 times.
Period of gestation not stated_____________45 times.

### Etiology.

The etiology of appendicitis in a pregnant woman is the etiology of appendicitis in the non-pregnant woman. It is the belief of many clinicians that gestation does not exert any influence, good or bad, upon the normal appendix.

Appendicitis is primary or secondary; it may be secondary to disease of the uterine adnexa just as inflammatory diseases of the tube and ovary may be secondary to an appendicitis. Recurrent attacks of appendicitis may be precipitated by pregnancy, labor or puerperium. Pregnancy can provoke acute inflammatory disturbances in an appendix bound down by dense adhesions or containing a foreign body, one or more concretions, or worms. The appendicitis complicating pregnancy may be the patient's first attack. It may have been preceded by one, two, three, or more attacks of greater or less severity.

**Combined Appendicitis and Extra-Uterine Pregnancy.**

In some of the reported cases in which appendicitis and ectopic pregnancy were associated, it was not determined which of the two conditions antedated the other; which was primary and which was secondary.

When an appendicitis precedes a tubal pregnancy in which it apparently plays an etiological role, the anatomical changes frequently evolute as follows:

1. Appendicitis.
2. Peri-appendicitis.
3. Peri-adnexitis.
4. Formation of inflammatory adhesions interfering with tube mobility and tube function and producing tubal malformation.
5. Tubal pregnancy.

All these conditions favor the ectopic implantation of fertilized ova. Appendicitis may hasten tubal abortion through local infection, through general intoxication, may lead to suppuration of hematoceles of festal cysts.

To differentiate appendicitis from extra-uterine pregnancy is at times difficult. In the unruptured state, the pregnant tube gives symptoms analogous to those of chronic appendicitis. An infected hematocele presents the signs of suppurative pelvic peritonitis. Peritoneal hemorrhage due to a ruptured tubal gestation sac has symptoms closely resembling a diffuse septic peritonitis. Positive Abderhalden test, absence of fever, vaginal hemorrhage, symptoms of internal hemorrhage will point to tubal pregnancy. It is interesting to make an exact diagnosis but as both diseases are surgical affections exposing mother and fetus to serious danger, the watchword in both conditions should be early operation. Appendicitis calls for prompt operative treatment; extra-uterine pregnancy is an emergency condition calling for immediate ablation of the ectopic fetal sac.

In all the cases of appendicitis and extra-uterine pregnancy herein considered, twelve in number, operation gave excellent results. The findings differed in nature and consequently the operative procedures varied in extent in the different cases.

### Pathology.

Acute and chronic inflammations of the appendix involve the organ, in part or in its entirety, and are associated with catarrhal, fibrinous, sero-fibrinous, sero-purulent, or purulent exudates present in the cavity of the appendix, in its wall, or around it. The inflammatory process may be limited to the mucous membrane, may involve part of, or the entire thickness of the appendical wall.

The appendix vermiformis may be partly or wholly intra- or extra-peritoneal. A retro-peritoneal or extra-peritoneal appendix the seat of suppurative inflammation gives rise to retro-peritoneal or extra-peritoneal pus collections. Adhesive inflammation may lead to permanent fixation of the appendix, to one or more abdominal viscera normal or pathologic, to the abdominal parietes, or to both. Inflammatory adhesions involving the tube, may angulate it, constrict it; may interfere with tubal mobility and tubal function; may change its course and play a fairly important role in the etiology of sterility. The appendix, during a 280-day pregnancy, may touch every organ of the abdomen. Pus in quantities, large or small, may be present within the cavity of the appendix, in its wall or around it. Acute suppurative inflammation of the uterus and tubes may be set up by direct extension from an acutely inflamed appendix. The walls of appendiceal or peri-appendiceal abscesses are formed in part by one or more of the following organs: uterus, adnexa, omentum, intestine, small or large, etc. An appendicular abscess may bulge into the posterior cul-de-sac, may open spontaneously into the uterus, vagina, rectum.

The inflammation proceeded to the state of gangrene in twenty-four cases; in eleven of these cases one or more perforations were present. The gangrene may be limited to the mucous membrane, may affect the entire appendiceal wall or the entire organ. Any part of the organ, tip, middle, base, may be gangrenous. Fecal concretions, one or more, were present in thirteen appendices. It is easy to understand how inflammation migrates from the appendix to the Fallopian tube, to the pregnant uterus, etc. Pelvic inflammatory processes extending by continuity or contiguity of tissue, occur in the pregnant as well as the non-pregnant. Distal pus collections are due to metastases by way of the lymph or blood channels. In the ulcerative type of inflammation the ulcer extends in depth and in surface area; when all the coats of the appendix have been burrowed through, a perforation results· The apex, the base, or any other part of the appendix may be the seat of perforation.

### Co-existing Pathological Conditions.

Co-existing pathological conditions are primary or secondary to the appendiceal inflammation or merely coincidental, bearing no relation of cause or effect to it· It is not uncommon for appendicitis in the female to be complicated by or associated with tubal and ovarian diseases: salpingitis, pyosalpinx, hydrosalpinx, ovarian abscess, tube-ovarian abscess, parametritis, etc. Close anatomical association of the appendix with the uterus and the adnexa explains the frequent simultaneous involvement of these organs in disease processes.

### Influence of Pregnancy Upon Appendicitis.

Upon a normal appendix, gestation has little or no influence. Upon an appendix, the seat of previous or latent disease, pregnancy exerts an unfavorable influence. It can intensify an existing inflammation. It may cause a previous inflammation to recur. In view of this possibility, many of our best clinicians recommend and practice the removal of the appendix in women married or about to be married who have had one or more attacks of appendicitis non-operatively treated.

The pregnant uterus as it ascends in the abdomen commonly displaces the coecum and the appendix from below up, from right to left and from behind forward. In enlarging, the uterus may stretch existing inflammatory adhesions; it may displace, twist, and kink the appendix and thereby whip into activity latent appendicular infections. Pregnancy is a serious complication of appendicitis. 1. When the appendix is adherent to the uterus. 2. When it is the seat of an inflammation, perforative, gangrenous or suppurative in type. 3. When its inflammation leads to abscess formation, near or distal. 4. When the uterus forms part of the wall of an appendicular, peri- or para-appendicular abscess. In the aforementioned conditions, adhesions may be torn, abscesses may be ruptured by the enlarging uterus.

### Influence of Appendicitis on Pregnancy.

Appendicitis is a menace to the mother's life, it is a menace to the gestation. The danger increases with the advance of gestation and is most marked after the fourth month. Infection can and does spread from the appendix to the genital organs by way, 1. Of the peritoneum (localized or diffuse peritonitis). 2. Of the appendiculo-ovarian ligament. 3. Of adhesions existing between the uterus and a perityphilitic pus focus. 4. Of the Fallopian tube.

Even a mild case of appendicitis may lead to a plastic peritonitis closing permanently the lumina of both tubes. From inflammatory adhesions may result dysmenorrhea, subinvolution, sterility through inflammatory closure of tubal ostia, habitual abortion, extra-uterine pregnancy, a tendency to uncontrollable vomiting, etc.

Appendicitis in the pregnant state may or may not terminate pregnancy. The prognosis is good as to non-interruption of pregnancy. 1. When the appendix does not hang in the small pelvis. 2. When the inflammation is limited to the appendiceal mucosa· 3. When it does not extend beyond the appendiceal wall. 4· When the appendiceal abscess or periappendiceal abscess is small.

Premature termination of gestation either by fetal death, fetal expultion or both may be caused by: 1. Sequels of previous appendicitis, acute or chronic; inflammatory adhesions, old or recent, preventing uterine expansion. 2. Infection from the appendix extending through the tubes to the uterus and its contents. 3. Infection reaching the placenta through lymphatic and vascular channels. 4. Metastatic inflammation of the placenta disturbing its circulation. 5. Local irritation. 6. Fatal effect of hyperpyrexia upon ovum.

The further pregnancy is advanced the greater the danger of abortion after operation. The chance of abortion after early operation is very small indeed for the operation is then done before an extensive inflammation has involved the uterus or an abscess rendered the patient septic. Tendency to abortion is small in clean cases as in this type the operative manipulation is reduced to a minimum.

In 173 cases of appendicitis herein studied it is stated that abortion was artificially induced nine times and occurred spontaneously forty-nine times. Caesarian section was performed four times· Abdominal one, vaginal, three·

In eighty-three cases, pregnancy was not interrupted by the operation. In seventeen cases, no definite statement is made.

### Diagnosis.

Appendicitis is not as frequently misdiagnosed as formerly. Increased familiarity with the condition enables us to make an earlier and a more timely diagnosis. It is an established fact that the morbidity and mortality of this disease can be lessened if it be diagnosed and operated, before the advent of complications, perforation, gangrene, abscess formation, peritoneal involvement, etc. The diagnostic difficulties increase with the advance of gestation and persist during the puerperium.

The symptomatology of appendicitis in the pregnant is the symptomatology of the disease in the non-pregnant. Nevertheless, the recognition of the condition is made more difficult by various factors. One or more of the carinal symptoms may be lacking. The symptoms and signs may not be sufficiently pronounced to lead to careful investigation or may be classed among the various disturbances incident to pregnancy.

During pregnancy the abdominal walls are on the stretch; they lack the softness and pliability so essential to careful and satisfactory abdominal palpation. In very fleshy patients, palpation does not give definite findings.

The seat of pain though always corresponding to the site of the inflamed appendix, may be abnormally high. The leukocyte count gives uncertain findings; at best, it has only relative or corroborative value.

Mistakes are less likely to occur by keeping in mind (a) that every pregnant woman is to be examined for physical defects. (b) That the history is all important; ask about previous attacks. (c) In gravid women, all attacks of indigestion associated with vomiting and fever should arouse suspicion and command a careful examination of the abdomen. (d) Right iliac pain unassociated with uterine contractions should lead one to think of appendicitis. (e) Deep seated retro-coecal and other abcesses may be detected by rectal examination. (f) Peri- or paratyphlitic abscesses may be detected by vaginal examination.

In a pregnant woman, acute abdominal pain of a sudden onset, at first diffuse and then remaining localized to the right iliac fossa, suggests appendicitis; more so if the patient gives the history of previous attacks.

### Differential Diagnosis.

During gestation, many conditions simulate appendicitis. As most of these conditions demand operative relief, the resulting diagnostic mistakes are embarrassing and humiliating to the surgeon, but not commonly disastrous to the patient. In adnexal disease the pain and the objective findings are most always bilateral, while in appendicitis they are unilateral and the pain as a rule is more acute. Non-ruptured right tubal pregnancy simulates and is frequently diagnosed chronic appendicitis. Rigidity and tenderness over McBurney's point are seldom marked in extra-uterine pregnancy. Intelligent interpretation of the clinical history and of the objective findings, furnished by a careful and thorough abdominal, rectal and vaginal examination helps one to arrive at a correct diagnosis. Abscesses in the pouch of Douglas due to perforative appendicitis have been wrongly attributed to primary uterine and tubal infection; right-sided parametritis due to the spreading of a retro-colic appendicitis has been diagnosed ordinary puerperal infection.

In pyelitis, uteritis, ureteric calcus of the right side one is guided by the urinary symptoms and findings. Hepatic colic has a sudden onset with pain in the right upper abdominal quadrant; this pain radiates toward the right shoulder and is usually apyretic. The pain of nephritic colic descends and rediates toward the external genitalia. In fecal impaction, the symptoms are less severe and yield to colonic injections and to laxatives.

In advanced pregnancy, the differential diagnosis between appendicitis and cholecystitis may prove difficult owing to the associated upward displacement of the coecum and appendix by the pregnant uterus.

### Prognosis.

Pregnancy increases the severity and the fatality of appendicitis. Death may be due to intestinal obstruction, to perforation of the appendix, to heart failure, to peritonitis, or to sepsis. Recovery takes place through the gradual subsidence of symptoms; through the spontaneous rupture of an appendicular abscess externally, or into the gut, vagina, urinary bladder, uterus, or other hollow viscus.

The type and the acuity of the inflammation influence the prognosis. The prognosis is good if the changes in the appendix are slight, if the inflammation is limited to the appendiceal wall; if there be slight or no peritoneal involvement, if complications be absent. It is grave in gangrenous, perforative and suppurative appendicitis and in all cases complicated by abscess formation, near or distal, or by diffuse peritonitis. The results for the mother and fetus are better, the less advanced the gestation, the less virulent and wide-spread the inflammation, the earlier the operation. Maternal mortality of appendicitis in pregnancy increases from the fourth month on.

As far as the child is concerned, prognosis is absolutely good in cases of early operated appendicitis. Severe maternal appendicitis is exceptionally grave for the fetus, who succumbs either through infection or through interruption of pregnancy. In our cases, there were fifty-eight abortions; of these nine were induced and forty-nine were spontaneous. The spontaneous abortions gave seventeen maternal deaths and thirty-two recoveries. The induced abortion gave four maternal deaths and five recoveries.

### Prophylaxis.

The cause of appendicitis is not known. Therefore, in the present state of our knowledge a discussion of the prophylaxis of appendicitis, of necessity, must be and is incomplete, inadequate and inconclusive. The importance of constipation as an etiological factor in appendicitis is as yet undetermined. We do not know how to prevent appendicitis, but we do know how to lessen its morbidity and mortality. Some surgeons remove the appendix during the course of all laparotomies. The removal of a healthy organ because one is not certain that it will always remain free of disease is unnecessary, meddlesome, and contrary to the teachings of conservative surgery.

In all laparotomies for conditions other than appendicitis, if the patient's condition permits, the appendix should be examined and removed: 1. If it be abnormal in length, size or location. 2. If it be in close relation to a pedicle or denuded surface, left by operation. 3. If its cavity be partly or wholly obliterated. 4. If it be the seat of anatomic alterations, club-shaped, thickened, kinked, twisted, strictured, etc· 5. If it contain foreign bodies, fecal concretions, worms, etc. 6. If it be adherent, in part or in its entirety, to some normal or diseased contiguous organ or to the abdominal parieties. 7. If it be the sole content or one of the contents of a hernial sac. 8. If it be the seat of cystic, neoplastic or inflammatory disease.

Operations that contribute to the safety of a pregnant woman should be performed without hesitation.

(To be Continued)

